# Preparation and Mechanical Properties of (TiCrZrNb)C_4_-SiC Multiphase High-Entropy Ceramics

**DOI:** 10.3390/ma17236024

**Published:** 2024-12-09

**Authors:** Chunmei Wen, Shuai Xu, Bingsheng Li, Shuyun Gan, Li Wang, Keyuan Chen, Yuwen Xue, Zhongqiang Fang, Fan Zhao

**Affiliations:** 1 School of Materials and Chemistry, Southwest University of Science and Technology, Mianyang 621010, China; wcm021515@163.com (C.W.); 13689673376@163.com (S.G.); cky_1999@163.com (K.C.); xueyuwen0519@163.com (Y.X.); 2 State Key Laboratory of Environment-Friendly Energy Materials, Southwest University of Science and Technology, Mianyang 621010, China; libingshengmvp@163.com (B.L.); 18728569050@163.com (L.W.); 3 The First Research Institute of Nuclear Power Institute of China, Chengdu 610005, China; fangnpic@163.com; 4 School of Manufacturing Science and Engineering, Southwest University of Science and Technology, Mianyang 621010, China; fzhao@swust.edu.cn

**Keywords:** ball milling process, high-entropy ceramics, microstructure, mechanical property

## Abstract

Five carbide powders, TiC, Cr_3_C_2_, ZrC, NbC and SiC, were selected as raw materials and mixed by dry or wet milling. Then (TiCrZrNb)C_4_-SiC multiphase ceramics were successfully prepared by spark plasma sintering (SPS) at 1900 °C, using D-HECs-1900 (dry milling method) and W-HECs-1900 (wet milling method), respectively. In this study, the effects of the ball milling method on the microstructure and mechanical properties of the multiphase high-entropy ceramics were systematically investigated. Compared to D-HECs-1900, W-HECs-1900 has a more uniform elemental distribution and smaller grain size, with an average grain size of 1.8 μm, a higher Vickers hardness HV_0.1_ = 2178.41 kg/mm^2^ and a higher fracture toughness of K_IC_ = 4.42 MPa·m^1/2^. W-HECs-1900 also shows better wear resistance with a wear rate 1.01 × 10^−8^ mm^3^·N^−1^·m^−1^. The oxide friction layer formed during friction is beneficial for reducing frictional wear, making W-HECs-1900 a potential wear-resistant material.

## 1. Introduction

Inspired by high-performance high-entropy alloys, high-entropy ceramics (HECs), including high-entropy oxides, borides, silicides, nitrides, and carbides, have been extensively researched due to their unique properties. At present, the research on HECC is still in its early stages. Compared to single transition metal carbides, HECC has higher elastic modulus and hardness [1], which facilitates its application in wear-resistant environments.

For ceramics, the higher the hardness, the more brittle the material. In order to enhance the fracture toughness of HECC, SiC particles and fibers are usually employed as effective secondary phases to improve the fracture toughness of transition metal carbides [2,3,4,5,6]. Kuan Lu et al. [7] used spark plasma sintering (SPS) to prepare (Ti_0.2_Zr_0.2_Hf_0.2_Nb_0.2_Ta_0.2_)C ceramics reinforced with 20 vol% SiC particles. The results showed that the introduction of SiC particles promoted the sintering densification process and solid solution formation, and significantly suppressed the grain growth of the high-entropy carbide phase. The mechanical properties, such as Vickers hardness, flexural strength and fracture toughness, were improved. After adding SiC particles, the fracture toughness of the sample sintered at 1900 °C increased from 4.51 MPa·m^1/2^ to 5.24 MPa·m^1/2^, which was mainly attributed to the crack deflection caused by SiC particles. Si-Chun Luo et al. [8] used silicon carbide whisker (SiC_w_) to toughen high-entropy ceramics. The results showed that the fracture toughness of ceramics reached 4.3 MPa·m^1/2^ after adding 20 vol% SiC_w_, which was 43% higher than that of high-entropy ceramics without SiC.

In this study, TiC, Cr_3_C_2_, ZrC, NbC and SiC powders were selected to prepare (TiCrZrNb)C_4_-SiC multiphase high-entropy ceramics by spark plasma sintering. By adjusting the ball milling process (ball milling time, ball milling medium) and sintering parameters (temperature, pressure, time), the effects of different ball milling processes on the microstructure, mechanical properties and tribological properties of sintered ceramics were investigated.

## 2. Materials and Methods

### 2.1. Processing

In this experiment, carbide powders were used as raw materials, including TiC (purity 99%, particle size 6~10 μm, Aladdin Biochemical Technology Co., Ltd., Shanghai, China), SiC (purity 99%, Shanghai Aladdin Biochemical Technology Co., Ltd., China), NbC (purity 99%, particle size 1~4 μm, Aladdin Biochemical Technology Co., Ltd., Shanghai, China), ZrC (purity 99%, particle size 1 μm, Shanghai Aladdin Biochemical Technology Co., Ltd., China), and Cr_3_C_2_ (purity 99.5%, Shanghai Aladdin Biochemical Technology Co., Ltd., China). Firstly, five kinds of carbide powders were mixed according to the molar ratio of cations, and 150 mL of absolute ethanol was added as the ball milling medium. The powder obtained was called wet grinding ceramic powder (W-GCP). At the same time, the powder obtained by ball milling without adding any medium is called dry grinding ceramic powder (D-GCP). The ball milling process is as follows: the rotation speed is 200 r/min, and the total ball milling time is 10 h, in which the ball milling is interrupted for 1 h after 5 h, and then continued for another 5 h. The mass ratio of powder to ball milled ball (ZrO_2_) was 1:5, and the wet milled ceramic powder was dried in an oven at 60 °C for 24 h. Subsequently, the dried W-GCP and D-GCP were placed in a graphite mold, respectively. Densification was carried out at 1900 °C and 50 MPa using SPS equipment (LABOX-650F, Sinterland, Nagaoka, Japan). The heating and cooling rates were set at 100 °C/min for 15 min. Cylindrical sample ceramic blocks with a diameter of 15 mm and a thickness of 8 mm were prepared and named D-HECs-1900 (dry grinding method) and W-HECs-1900 (wet grinding method). Finally, the sintered samples were cut into 7 × 5 × 1 mm^3^ rectangular blocks with a grinder wheel and mirror polished using a 1 μm polishing solution. This was used for subsequent characterization, relevant mechanical property tests and friction and wear tests.

### 2.2. Characterization

Measuring the Particle Size Distribution of Ground Powders Using a Laser Particle Size Analyzer (Ls 13,320, Beckman Coulter Co., Ltd., Brea, CA, USA). The micromorphology of the mixed powders was characterized by scanning electron microscopy SEM (TM-4000, Hitachi Limited Co., Ltd., Tokyo, Japan). Grain size was observed using electron backscatter diffraction (EBSD) (Nordly max3, Oxford Instruments Co., Ltd., Abingdon, UK), 1000 × 1000 grating, 0.15 μm step size, and parameter settings: acceleration voltage 20.00 kV, sample tilt 69.99°, and acquisition speed 318.65 Hz.

The phase structures and lattice parameters of the mixed powders and ceramics were determined by X-ray diffraction (Smartlab, Rigaku Corporation Co., Ltd., Tokyo, Japan), using Cu-K_α_ radiation, scanning angles ranging from 5° to 80°, and scanning steps of 0.02°. The XRD data was analyzed and processed by Jade 9 software. The bulk density ($\rho_{b}$) of the ceramic block was measured by the Archimedes drainage method, and the theoretical density ($\rho_{T}$) was calculated by the mixing rule. The relative density ($\rho_{r}$) was calculated according to Equation (1):

(1) $$\rho_{r}=\frac{\rho_{b}}{\rho_{T}}\times 100\%$$

The Vickers hardness(HV-1000, Shanghai Precision Instrument Co., Ltd., Shanghai, China) of the sample was tested using a microhardness tester. Before testing, the samples were ground and polished like a mirror. Subsequently, the Vickers hardness was tested under a load of 0.981 N (i.e., 0.1 kgf) with no visible cracks at the corners of the indentation, and the average Vickers value was obtained by 5 hardness measurements.

The internationally recognized indentation method was used to evaluate the fracture toughness of ceramic samples. The test procedure was the same as the Vickers hardness test procedure with a test force of 4.903 N (i.e., 0.5 kgf) and a holding time of 15 s. Because the cracks in the high entropy carbide ceramics in this thesis belong to the Palmqvist crack system, the fracture toughness, K_IC_, of the ceramic samples can be calculated by using the formula of James Lankford [9]:

(2) $$\left(\frac{K_{IC}\Phi }{{Ha}^{\frac{1}{2}}}\right){\left(\frac{H}{E\Phi }\right)}^{\frac{1}{2}}=0.142{\left(c/a\right)}^{-1.56}$$

**Figure 1:**
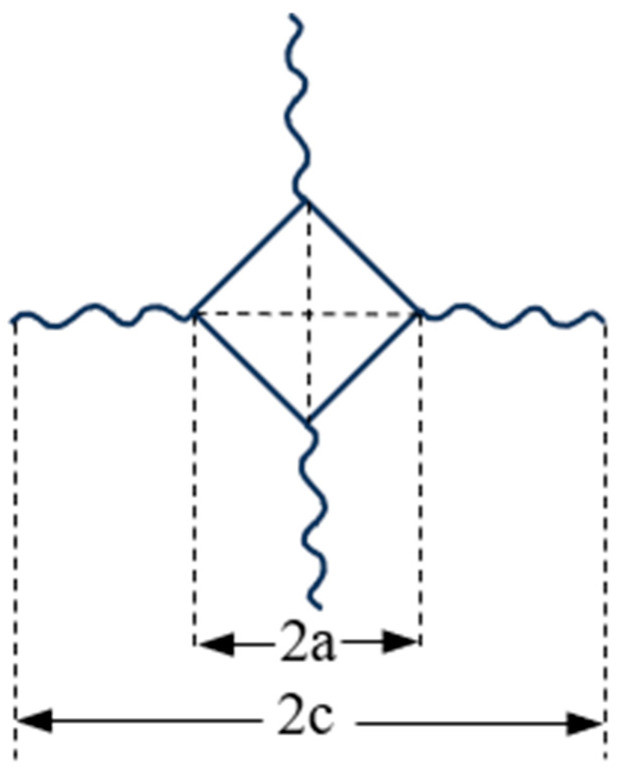
Characteristic lengths of the Vickers indentations.

In the equation, *H* is the Vickers hardness (GPa), *H* = 463.6 P/a^2^, *E* is the elastic modulus (GPa), P is the applied load (N), a and c (µm) are shown in Figure 1, and Φ is the constraint factor (≈3). Since the elastic modulus of (CrNbTiZr)C_4_-SiC multiphase high-entropy ceramics has not been reported, the elastic modulus of (CrNbSiTiZr)C in Wang Junjun’s study is 218.7 GPa [10], which is considered as the elastic modulus of (CrNbTiZr)C_4_-SiC multiphase high-entropy ceramics. Combined with the data related to hardness testing, where c/a < 2.5, the crack is a Palmqvist crack [11].

The friction tests were carried out using a friction wear tester (Rtec MFT-3000, Rtec Instruments Co., Ltd., San Jose, CA, USA). The pin-on-disk contact and reciprocating motion modes were performed at room temperature, and the friction pair was Al_2_O_3_. The sample size of the material was 7 × 5 × 1 mm^3^. The load was 15 N, the sliding speed was 3 mm/s, and the test time was 30 min. The wear rate was calculated by Equation (3):

(3) $$W=\frac{V}{F\times L}$$

where *W* is the wear rate (mm^3^·N^−1^·m^−1^), *V* is the wear volume (mm^3^), *F* is the load (N), and *L* is the friction sliding distance (m).

After friction tests, the microstructure and element distribution of the worn surfaces of HECs and HECs were characterized by field emission scanning electron microscopy (ULTRA55, Carl Zeiss AG Co., Ltd., Oberkochen, Germany).

## 3. Results and Discussion

### 3.1. Characterization of Ball Milled Powders

**Figure 2:**
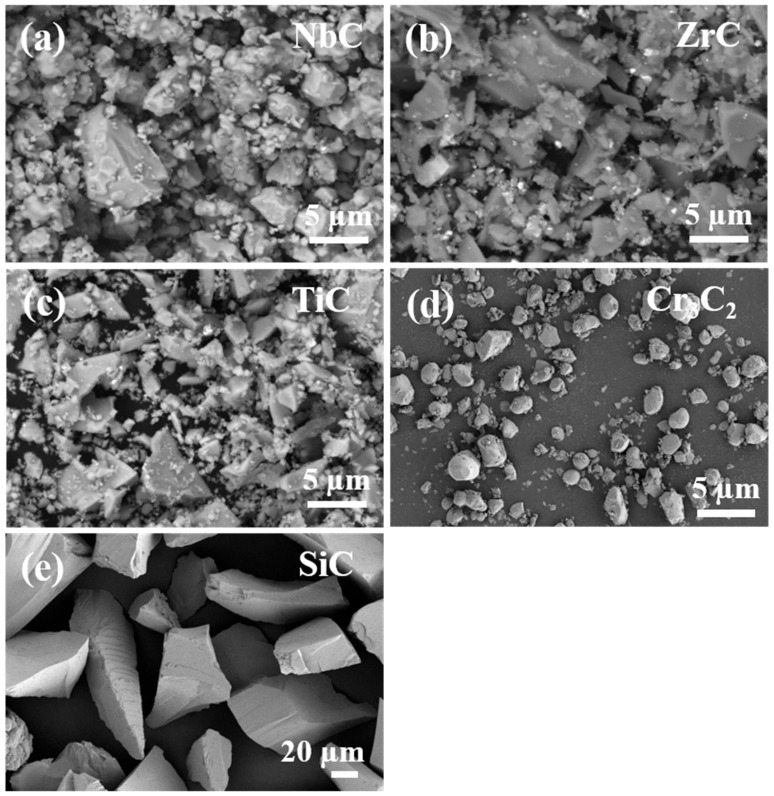
SEM images of NbC (**a**); ZrC (**b**); TiC (**c**); Cr_3_C_2_ (**d**); SiC (**e**).

**Figure 3:**
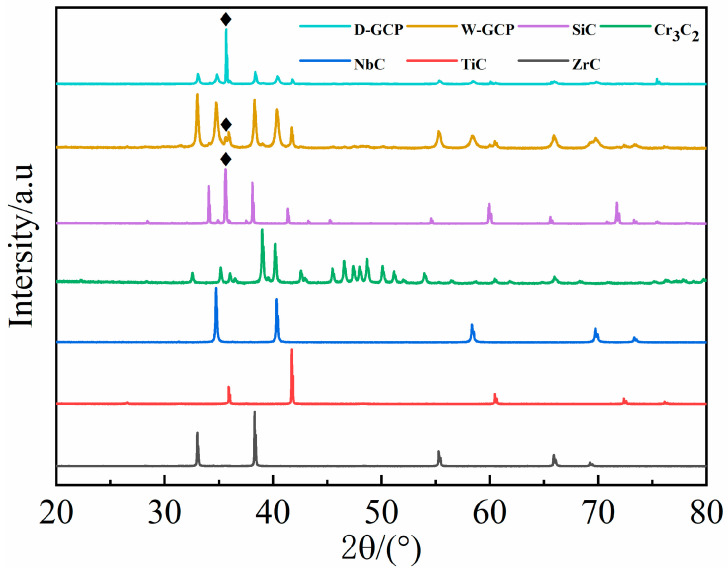
XRD patterns of W-GCP, D-GCP and carbide powders.

**Figure 4:**
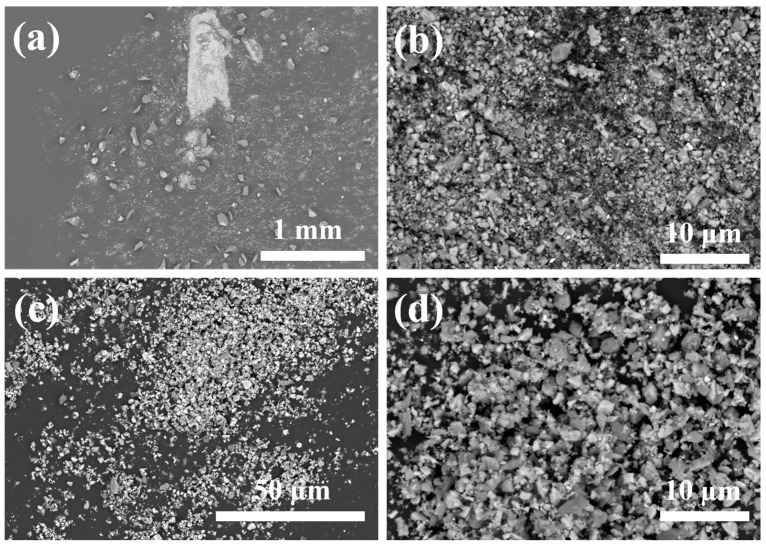
SEM images of D-GCP (**a**,**b**) and W-GCP (**c**,**d**).

**Figure 5:**
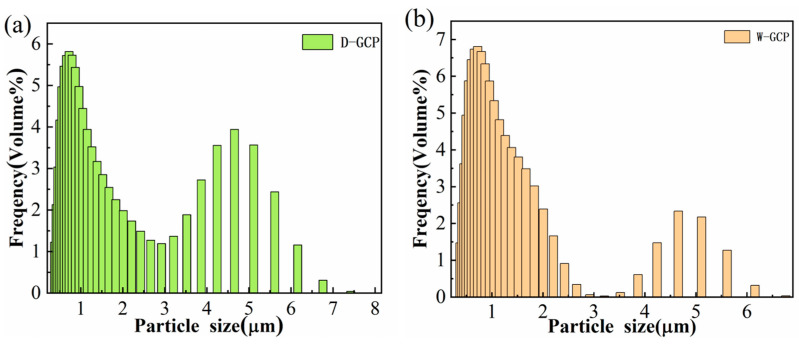
Particle size distribution histogram of D-GCP (**a**) and W-GCP (**b**).

Figure 2 shows SEM images of the original powder NbC, ZrC, TiC, Cr_3_C_2_, and SiC, where Cr_3_C_2_ has the smallest particle and SiC has the largest particle. Figure 3 shows the XRD pattern comparison of W-GCP, D-GCP and carbide powders. The mixed powders obtained by ball milling under different processes exhibited TiC (PDF#04-008-0231, space group: Fm$\bar{3}$m, a = b = c = 4.327 Å), ZrC (PDF#97-061-9158, space group: Fm$\bar{3}$m, a = b = c = 4.699 Å), NbC (PDF#04-002-0485, space group: Fm$\bar{3}$m, a = b = c = 4.469 Å), Cr3C2 (PDF#97-061-7482, space group: Pnma, a = 5.524 Å, b = 2.828 Å, c = 11.466 Å), and SiC (PDF#04-002-9070, space group: F$\bar{4}$3 m, a = b = c = 4.348 Å, β-SiC. PDF#04-007-1548, space group: P63mc, a = b = 3.081 Å, c = 15.117 Å, α-SiC; the weight ratio of α-SiC and β-SiC is 99.7:0.3). No diffraction peaks of other phases appear in the patterns. This shows that, under these experimental conditions, the energy provided by ball milling is not enough to cause the phase transformation of carbide powders, i.e., no new phase is formed and no reaction occurs between carbide powders. The observation results are consistent with those of Hao Chen et al. [12]. They performed a 10-h ball milling in a cemented carbide tank at a speed of 400 r/min, and then placed the mixed slurry in a drying oven and dried at a temperature of 80 °C for 12 h. In Figure 3, the diffraction peak of SiC phase in the D-GCP is significantly stronger than that in the W-GCP, as marked by the diamond symbol in Figure 3. This difference arises mainly due to the fact that the particle size distribution of the D-GCP is not as uniform as that of the W-GCP. In the D-GCP, there are many coarse-grained SiC particles (10–30 μm), as shown in Figure 4, and these particles are more easily detected in the XRD test. The fundamental reason for this phenomenon is that SiC has high hardness, and the particles are not easy to refine during dry grinding. Figure 4 shows the SEM images of D-GCP and W-GCP. It can be seen that D-GCP and W-GCP present many submicron-sized particles. In Figure 4a,b, the D-GCP has obvious large particles, the small particles are attached to the surface of the large particles, and there is agglomeration between the small particles. This is due to the decrease in particle size and the increase in surface area during ball milling, which leads to the enhancement of surface tension and activity, making it easy to cause agglomeration between particles. In contrast, the particle size after wet grinding in Figure 4c,d is more uniform and there is no agglomeration. The results of the LS particle size analyzer are the powder frequency distribution histogram are shown in Figure 5. Figure 5a,b shows a bimodal distribution, and both peaks are distributed in the nanometer region, whereas the finer powder in the W-GCP obviously has a higher volume fraction. Specifically, the statistical results show that the volume fraction of powder with particle size less than 3 μm in W-GCP accounts for 93%, while in D-GCP, the value is 79%. It should be noted that the volume fraction of powders with a particle size of less than 3 μm in dry grinding is less than 79% due to the limited test range of the LS particle size analyzer in detecting coarser particles. The average particle size of W-GCP is 1.30 μm, while that of D-GCP is 1.78 μm. This difference in particle size distribution is also verified in the SEM image of Figure 4. In summary, wet ball milling has a more desirable particle size distribution compared to dry ball milling. The reason is that in the early stage of wet ball milling, coarse particles produce a large number of cracks under the violent impact of ball milling beads, and some small particles enter the cracks to play the role of wedge cracking, promote crack propagation and accelerate particle fragmentation. In the later stage of ball milling, ethanol is wrapped on the surface of the particles, which effectively reduces the contact area between the particles, thereby reducing the chance of adsorption and agglomeration. It is worth mentioning that the powder particle size obtained in this experiment is much smaller than the carbide powder prepared by Jinyong Zhang et al. [13] using the wet milling method, and the carbide powder particle size prepared by ball milling for 24 h is 74 μm.

### 3.2. Microstructure Characterization and Analysis of Ceramics

**Figure 6:**
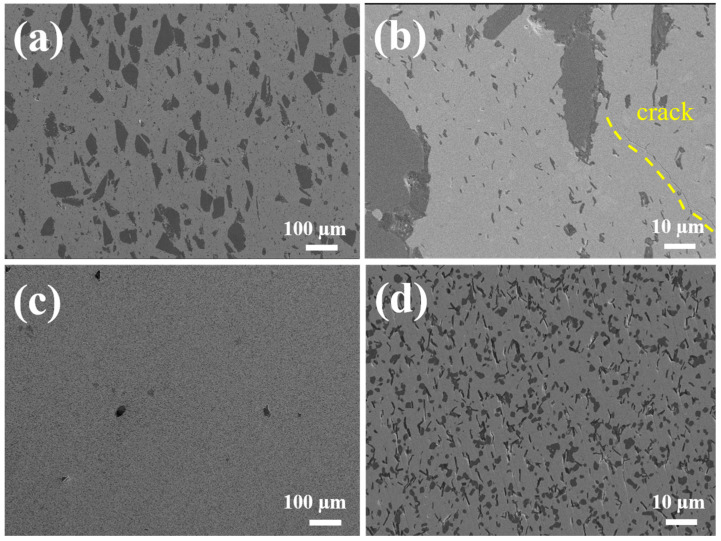
SEM image of D-HECs-1900 (**a**,**b**) and W-HECs-1900 (**c**,**d**).

**Figure 7:**
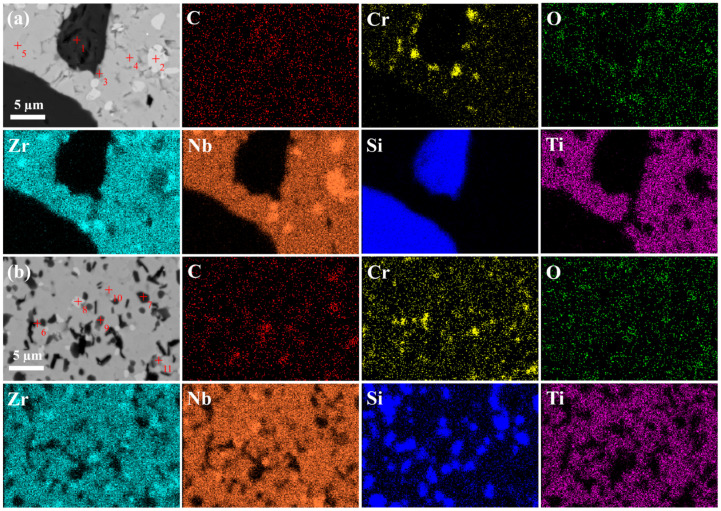
SEM images, point scans and element distribution mappings of D-HECs-1900 (**a**) and W-HECs-1900 (**b**).

The SEM images of the ceramics after polishing is shown in Figure 6. Under low magnification conditions, D-HECs-1900 shows a two-phase structure with light and dark contrasts, while W-HECs-1900 only shows a two-phase structure with light and dark contrasts under high magnification conditions, which is more homogeneous than that of D-HECs-1900 on the microscopic scale. It is worth noting that there are cracks over 100 μm long on the polished surface of D-HECs-1900 in Figure 6b, as shown by the dotted line, but this is not observed in W-HECs-1900. This phenomenon can be attributed to the non-uniformity of the powder particle size, which leads to non-uniformity of the internal stresses in the material after sintering, resulting in microcracks. As can be seen from Figure 7a,b, Ti, Cr, Zr, Nb, and Si elements are enriched to varying degrees. In order to further analyze the composition, the point scanning test was carried out on Figure 7a,b, and the results are shown in Table 1 and Table 2. Test points are marked with a red cross. Those marked as 1 and 7 are SiC. The points marked as 3, 4, 5, 6, 9, and 10, combined with subsequent EBSD data analysis, are (TiCrZrNb)C_4_. Marked as 2 is NbO_2_. Marked as 8 are SiC and (TiCrZrNb)C_4_. Marked as 11 is ZrO_2_. However, in the point scan data, the O content is much higher than that designed, which is inconsistent with the results analysed by XRD. The reason is that, in the point scanning test, the K line energy of C element (the energy generated by the electron transition from the L layer to the K layer) is 0.277 keV, and the K line energy of O element is 0.525 keV, so the two K line energies of C may be misjudged as O. Figure 6c is the SEM of W-HECs-1900, with obvious pores. In Figure 6d, SiCs with basically the same size can be seen in W-HECs-1900. These SiCs are evenly distributed in the high-entropy composite ceramic matrix. Detailed characterization shows that there are a large number of pores in the ceramics, some of which are trapped in the high-entropy phase, while most of the pores are located at the phase boundary between the high-entropy phase and the SiC. Compared with Figure 6b, the main reason why there is no crack in Figure 6d is that the particle size distribution prepared by the wet grinding method is more reasonable, which reduces the internal stress of the material.

**Figure 8:**
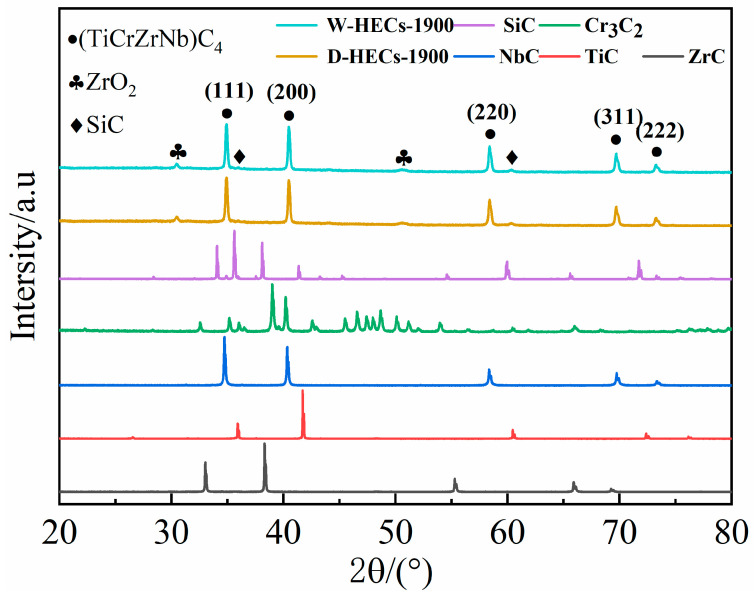
The XRD patterns of powders and sintered ceramics.

The X-ray diffraction pattern characterizes the grain structure of the ceramic, as shown in Figure 8. The results show that all the ceramics sintered by SPS exhibit the same phase structure, i.e., whether W-GCP or D-GCP, the phase obtained after SPS sintering is the same. Compared with the XRD patterns of the raw material powder, the number of diffraction peaks in the sintered ceramic is significantly reduced, indicating that the ball milled powder undergoes a solid solution reaction during the sintering process, forming an FCC structure (face centered cubic) [14,15]. Through Rietveld analysis, the lattice parameter of the main phase is obtained as 4.451 Å. There are some weak peaks in the XRD spectra of the ceramics, which are calibrated as SiC (PDF#04-010-5698, space group: P6_3_mc, a = b = 3.082 Å, c = 15.092 Å) and ZrO_2_ (PDF#04-006-1370, space group: Fm$\bar{3}$m, a = b = c = 5.100 Å). The SiC in the ceramic block is α-SiC, while the SiC in the mixed powder is α-SiC and β-SiC. The grain structure of SiC changes during sintering. The β-SiC is easily transformed into α-SiC at 2100 °C [16], while the phase transition occurs at a lower temperature in this experiment. This may be due to the refinement of SiC during ball milling and the pressure during sintering, which leads to the decrease in phase transition temperature. The existence of oxides is a common problem in the preparation process [14,17], which is mainly caused by raw material impurities and sintering atmosphere pollution. HfO_2_ and ZrO_2_ also exist in (Hf_0.2_Ta_0.2_Ti_0.2_Nb_0.2_Zr_0.2_)C high-entropy ceramics sintered by SPS in the method by Huifen Guo et al. [18]. In summary, TiC, ZrC, NbC, Cr_3_C_2_ and SiC are sintered into two-phase high-entropy ceramics by SPS. High-entropy carbide ceramics containing SiC in multiphase have been studied by Kuan Lu et al. [7], and Haoxuan Wang et al. [19] found a SiC phase in their synthesized high-entropy ceramics, but there is no discussion on the solid solution of SiC in the high entropy phase.

The Hume–Rothery solid solution rule suggests that the atomic-size effect (δ) and the enthalpy of mixing (ΔH_mix_) can be used as two main criteria to predict the formation of solid solutions. However, in the current case, ΔH_mix_ is difficult to quantify. Therefore, researchers focus their attention on analyzing the atomic size effect. In the Hume–Rothery solid solution rule ($r_{solute}-r_{solvent})/r_{solvent}\leq 15\%$, according to the same concept, the average atomic size difference (δ) of multi-component HEA alloys can be defined as [20]:

(4) $$\delta =\sqrt{\sum_{i=1}^{N}c_{i}{\left[1-r_{i}/(\sum_{i=1}^{N}c_{i}r_{i})\right]}^{2}}$$

where N is the total class of elements, ri and ci are atomic radius and molar content of component i.

Gild et al. [21] replaced r_i_ with the lattice constant of a single metal diboride (MB_2_) to calculate the average size difference of high-entropy metal diborides in order to evaluate the possibility of metal diboride solid solution formation. These high-entropy metal diborides exhibit a unique layered hexagonal structure, and the equation is as follows:

(5) $$\delta_{a}=\sqrt{\sum_{i=1}^{N}X_{i}{\left[1-a_{i}/(\sum_{i=1}^{N}X_{i}a_{i})\right]}^{2}}$$

(6) $$\delta_{c}=\sqrt{\sum_{i=1}^{N}X_{i}{\left[1-c_{i}/(\sum_{i=1}^{N}X_{i}c_{i})\right]}^{2}}$$

In the equation, *X*_i_ is the molar content, a_i_ and c_i_ are the lattice parameters a_i_ and c_i_ of the MB_2_, respectively. In this study, the calculated δ_c_ value can better represent the average size difference, so it is considered that δ_c_ ≤ 5.2% will form a simple high-entropy phase. On this basis, Beili Ye et al. [22] applied it to calculate the possibility of forming (Zr_0.25_Nb_0.25_Ti_0.25_V_0.25_)C high-entropy carbide solid solution:

(7) $$\delta =\sqrt{\sum_{i=1}^{n}c_{i}{\left[1-R_{i}/(\sum_{i=1}^{n}c_{i}R_{i})\right]}^{2}}$$

In the equation, n represents the type of metal carbide component in the multi-component metal carbide solid solution, c_i_ represents the atomic percentage of the *i*-th component of the metal carbide, and *R*_i_ represents the lattice constant of the metal carbide. The calculated δ = 4.59%, which is less than 5.2% in Gild’s literature, means that the possibility of forming (Zr_0.25_Nb_0.25_Ti_0.25_V_0.25_)C high-entropy carbide single-phase ceramics is considered. The information regarding the grain structure of the carbide powder raw material is shown in Table 3, and the results of calculations using Equation (7) in this paper are shown in Table 4.

In general, the smaller δ may help to reduce the lattice distortion and the corresponding strain energy in the system, and the possibility of forming a solid solution is higher. In Table 4, δ_a_ represents the average atomic size difference, in which the formation of (TiCrZrNb)C_4_ and β-SiC solid solution into (TiCrZrNb)C_4_ to form (TiCrZrNbSi)C_5_ are equally possible. Although Junjun Wang et al. [10] and Yu-Siang Jhong et al. [23] successfully prepared (TiCrZrNbSi)C thin films by magnetron sputtering, a higher sintering temperature is required for the solid solution of SiC into (TiCrZrNb)C_4_. Junjun Wang et al. [10] calculated that (TiCrZrNbSi)C is thermodynamically stable above 2485 °C using the first principle, but the sintering temperature of this experiment is 1900 °C, which does not reach the theoretical calculation value.

**Figure 9:**
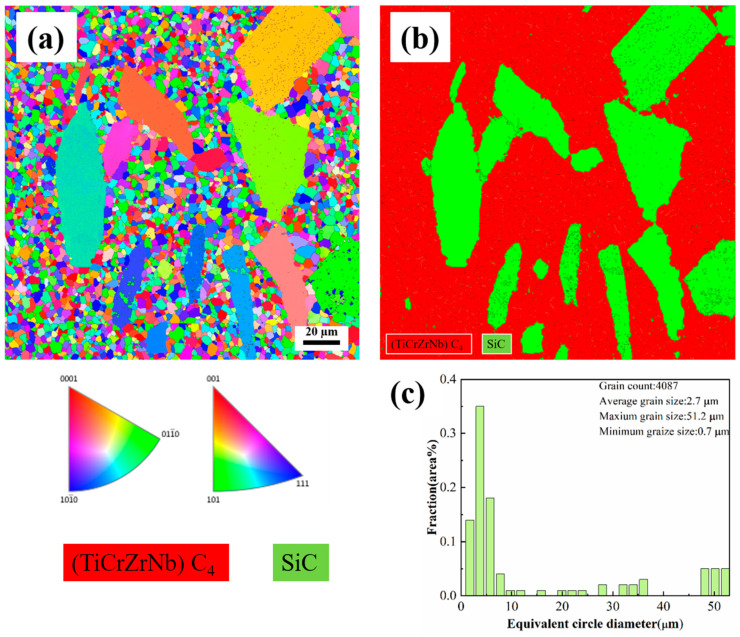
EBSD image of D-HECs-1900 (**a**); the corresponding EBSD phase distribution map (**b**); grain size distribution of D-HECs-1900 based on EBSD results (**c**).

**Figure 10:**
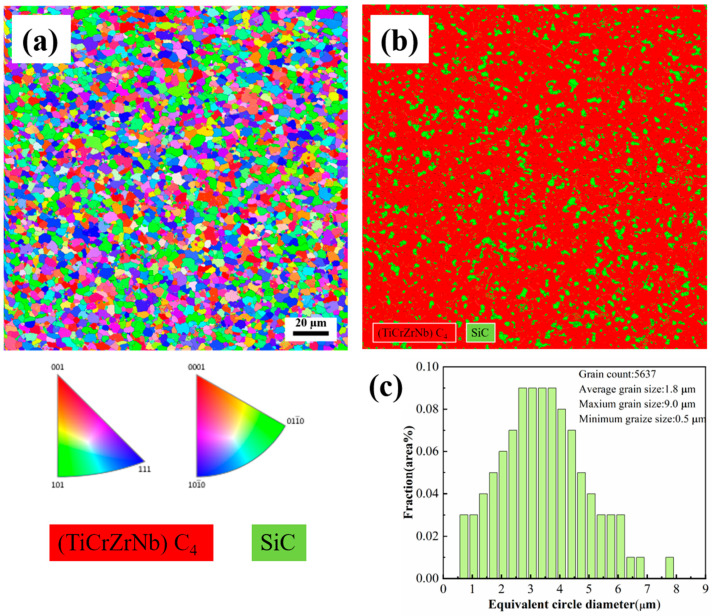
EBSD image of W-HECs-1900 (**a**); the corresponding EBSD phase distribution map (**b**); grain size distribution of W-HECs-1900 based on EBSD results (**c**).

The microstructure of the ceramic blocks was characterized by electron backscatter diffraction (EBSD, Bruker QUANTAX, Billerica, MA, USA) in field emission gun scanning electrons. Figure 9 is the EBSD diagram of D-HECs-1900. In Figure 9a, the grain orientation of different grains is determined by EBSD diffraction, revealing that the orientation of these grains is randomly distributed. Figure 9b shows the analysis results of the phase distribution diagram. The phases are mainly composed of cubic (TiCrZrNb)C_4_ high entropy phase (space group: Fm$\bar{3}$m) and trigonal SiC phase (space group:P6_3_mc), accounting for 63.03% and 27.31%, respectively. Figure 10 is the EBSD diagram of W-HECs-1900, and the proportion of high entropy phase and SiC phase is 77.53% and 12.87%. This is consistent with the SEM and XRD results mentioned above. Figure 9c shows the direct statistical results of EBSD data for D-HECs-1900. More than 4000 grains are used to calculate the particle size distribution. The average grain size is 2.7 μm, and the number of coarse grains with a grain diameter greater than 3 μm accounts for 87%. Figure 10c shows the grain distribution of W-HECs-1900, with a total of 5637 grains. The maximum grain size is 9.0 μm, the minimum grain size is 0.5 μm, the average grain size is 1.8 μm, and the number of coarse grains with grain diameter greater than 3 μm accounts for 62%. Using the spark plasma sintering method, the density should reach more than 99%. Usually, the sintering temperature is above 2000 °C, and the higher sintering temperature leads to a generally larger grain size (1–20 μm). In Table 5 [24,25,26,27,28,29,30,31], the grain size obtained in this study is smaller due to the lower sintering temperature; the added silicon carbide particles play a pinning role and hinder the grain growth [27]. W-HECs-1900 prepared by Qichun Sun et al. [29] and in this study used high-entropy powders synthesized by carbothermal reduction method as raw materials. The grain size (1.7 ± 0.6 μm) and density of (Hf_0.2_Mo_0.2_Nb_0.2_Ta_0.2_Ti_0.2_)C prepared by sintering were similar. Therefore, the density and grain size of high-entropy carbide ceramics prepared by SPS are not only affected by sintering process conditions, but also related to the properties of raw material powders, such as particle size, distribution, etc.

### 3.3. Characterization and Analysis of Mechanical Properties of Ceramics

**Figure 11:**
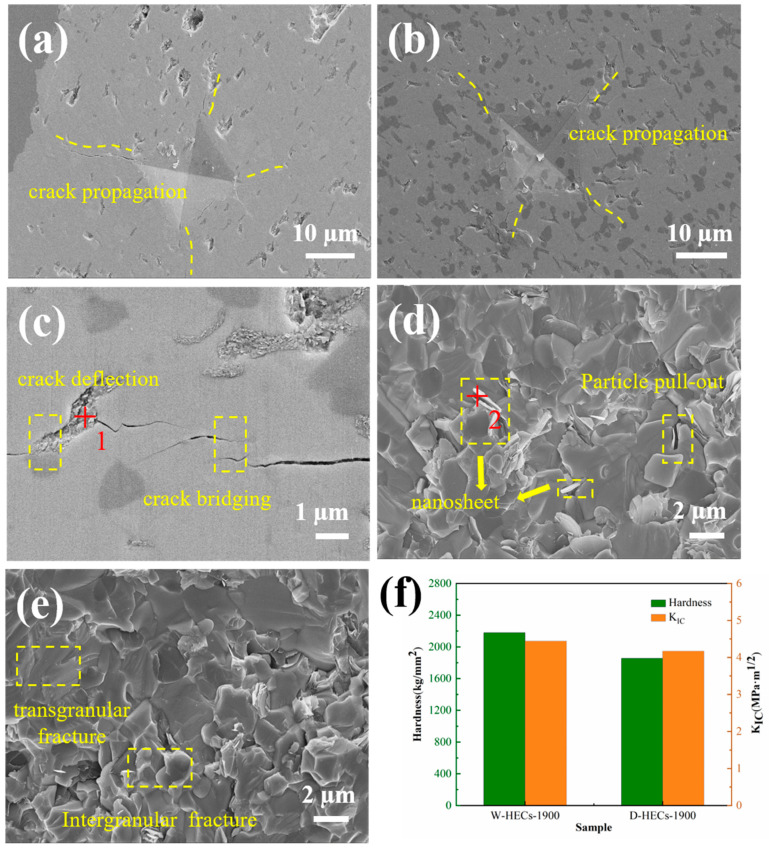
Vickers indentation SEM images of D-HECs-1900 (**a**) and W-HECs-1900 (**b**); Crack propagation diagram (**c**); section diagram (**d**,**e**); Comparison of hardness and fracture toughness (**f**).

According to the Hall–Petch [32] relationship, the hardness of ceramics generally decreases with the increase in grain size ($H=H_{0}+k^{\prime }d^{-1/2}$, where H is the hardness, d is the grain size, H_0_ and k′ are constants independent of grain size). The Vickers hardness test and indentation method were used to measure the fracture toughness of the high-entropy ceramics prepared by the two methods. The test results are shown in Figure 11f. The Vickers hardness values of D-HECs 1900 and W-HECs 1900 are HV_0.1_ = 1855.87 kg/mm^2^ and HV_0.1_ = 2178.41 kg/mm^2^. The fracture toughness of D-HECs 1900 and W-HECs 1900 are K_IC_ = 4.17 MPa·m^1/2^ and K_IC_ = 4.44 MPa·m^1/2^. The Vickers hardness and fracture toughness of W-HECs-1900 are higher than those of D-HECs-1900. The reason is that the high-entropy ceramics prepared by wet grinding have smaller grains and higher density. The morphology of the indentation area is characterized by scanning electron microscopy, as shown in Figure 11a,b. There are obvious cracks around the indentation of dry and wet ball milling ceramics. Although the number of cracks is large, the shape is relatively deflective, and the black part shows SiC particles. Figure 11c shows the indentation crack propagation diagram. Based on the composition analysis of point 1, according to the research into and description of amorphous carbon in the literature [18], it can be considered that this point is amorphous carbon. In Figure 11c, it can be clearly observed that deflection and bridging occurred during crack extension, and the SiC particles and amorphous carbon acted with a strong shielding effect crack extension. When the crack propagation meets with SiC particles and amorphous carbon, a force is generated to close the crack, and the crack consumes more energy to continue to expand, which shortens the length of crack propagation and improves the toughness of composite ceramics. During the cooling process of ceramic sintering, due to the mismatch of thermal expansion coefficient between SiC particles and ceramic matrix, the residual stress field is formed around the interface between SiC particles and ceramic matrix, and the interface bonding is weak. Under the influence of local tensile stress, microcracks will form on weak interfaces. When the main crack propagation encounters these microcracks, it will bifurcate and turn forward, increasing the surface energy during the propagation process. At the same time, the stress concentration at the tip of the main crack is relaxed, which slows down the propagation speed. These factors increase the toughness of the material. Figure 11d,e shows the microscope fracture surfaces. It can be seen that the fracture mode of ceramics is the combined action of trans-granular fracture and intergranular fracture. Irregular nanosheet structures with thickness of approximately 80 nm are observed at the fracture surface in Figure 11d, and their components are shown in Table 6, which indicates that these are carbon nanosheets. The obvious deflection of the crack improves the toughness of the material. The appearance of these carbon nanosheets is due to the higher enthalpy of mixing that separates the phases when forming a solid solution [22]. This phenomenon also exists in the (Zr_0.25_Nb_0.25_Ti_0.25_V_0.25_)C high-entropy carbide ceramics prepared by Beilin Ye et al. [22]. Their results show that the pull-out of nanosheets has a toughening mechanism. In the study of Sichun Luo et al. [15], it was also clearly mentioned that carbon-rich nanosheets can deflect cracks.

The fracture toughness of other high-entropy ceramics by the Vickers indentation method is summarized in Table 7 [26,33,34,35,36]. The results show that the fracture toughness obtained in this study using Equation (2) is higher than that in other literature. The reason may be the result of the joint action of the toughening mechanisms of microcracking, deflection and crack bridging. The reason for the high fracture toughness of W-HECs-1900 is that the SiC distribution is more uniform during the wet grinding process, forming more weak interfaces and further improving the fracture toughness.

**Figure 12:**
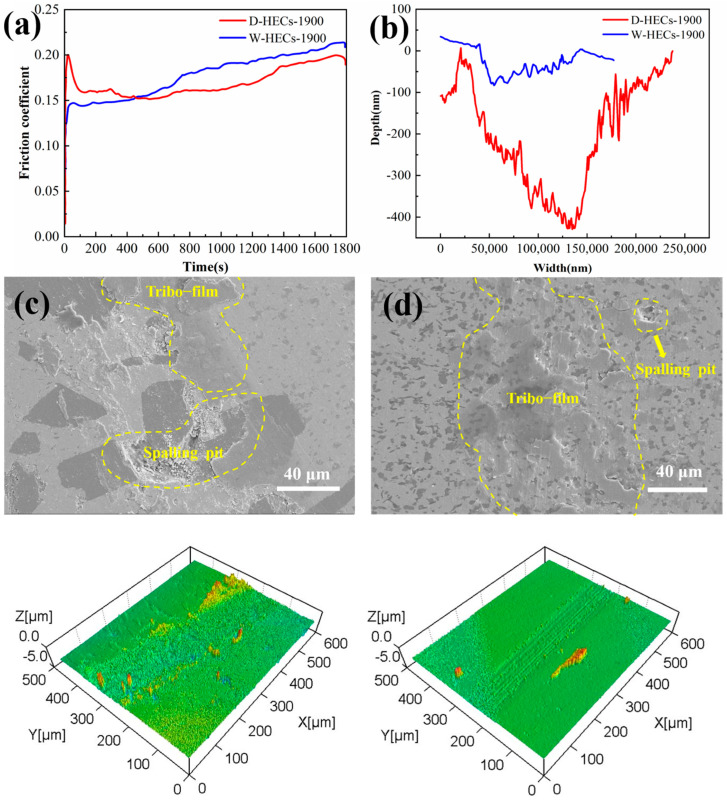
Friction coefficient comparison diagram (**a**); Wear trajectory profile (**b**); D-HECs-1900 wear surface and three-dimensional contour map (**c**); W-HECs-1900 wear surface and three-dimensional contour map (**d**).

**Figure 13:**
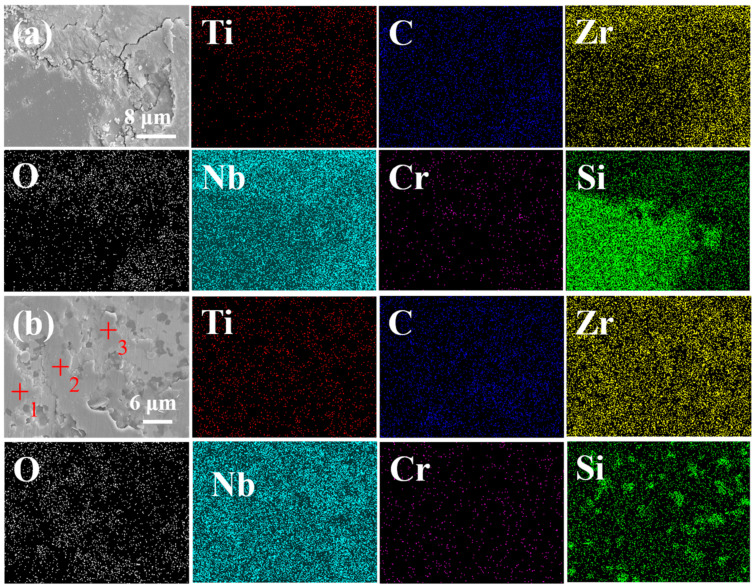
SEM images and element distribution mappings after friction wear of D-HECs-1900 (**a**) and W-HECs-1900 (**b**).

According to the literature [37], we see increased hardness and increased wear resistance. In order to further understand the mechanical properties of (TiCrZrNb)C_4_-SiC composite high-entropy ceramics, friction and wear experiments were carried out. From the experimental data, the wear volume of D-HECs-1900 is 1.17 × 10^−4^ mm^3^, and the wear rate is 1.44 × 10^−7^ mm^3^·N^−1^·m^−1^. The wear volume of W-HECs-1900 is 8.16 × 10^−6^ mm^3^, and the wear rate is 1.01 × 10^−8^ mm^3^·N^−1^·m^−1^. Wear resistance is the performance of the material in resisting wear, which is a systematic property and can be expressed by wear volume. The smaller the wear volume, the higher the wear resistance, i.e., compared with D-HECs-1900, W-HECs-1900 has higher wear resistance. It can be seen from Figure 12a,b that the friction coefficient of D-HECs-1900 is 0.18, and the friction coefficient of W-HECs-1900 is 0.22. The wear trajectory of W-HECs-1900 is smaller than that of D-HECs-1900. In this paper, the friction coefficient of the two materials is small, which is related to the addition of SiC particles. There are two reasons given in the studies of Guangyu Yu et al. [38]. On the one hand, the addition of SiC particles improves the hardness of the ceramic layer, effectively inhibits the fracture and spalling of the composite surface during the friction process and enhances its ability to resist wear. On the other hand, the SiC particles with good thermal conductivity also reduce the flash temperature between the friction pairs in the composite ceramics and reduce the adhesive wear between the friction pairs. Careful observation of Figure 12c,d shows that there is no obvious ploughing, but peeling pits and discontinuous friction layers are formed, which have the morphological characteristics of adhesive wear and abrasive wear. During the friction process, the material grains are broken to form wear debris, some of which is scattered on the surface of the material, and the other part of the wear debris is randomly or discontinuously involved in the contact area. After repeated friction, the particles are bonded to each other to form agglomerates and adhere to the surface of the composite material, forming a surface layer with a certain anti-wear effect. In order to understand the friction layer more deeply, the element analysis of the friction layer was carried out. The results of Figure 13 show that the elements of Ti,C,Zr,Nb,Cr and Si on the surface of the friction layer are evenly distributed, but the O element is enriched. Combined with the analysis of point scan data Table 8, this shows that the friction layer is oxidized, i.e., there is oxidative wear during friction and wear. This friction oxide film can reduce the friction coefficient and wear degree according to the literature [39,40].

## 4. Conclusions

In this study, the microstructure and mechanical properties of high-entropy ceramic samples prepared by dry ball milling and wet ball milling were analyzed. The following conclusions were obtained:

1. No matter which dry ball milling process or wet ball milling process, no new phase was formed during the ball milling stage. With absolute ethanol as the ball milling medium, the wet milling mixed powder obtained after ball milling for 10 h and the dry milling mixed powder obtained by ball milling for 10 h all show a bimodal particle size distribution, but the powder prepared by the wet ball milling has a higher degree of refinement, and the particle size distribution is more conducive to subsequent ceramic sintering.
2. The high-entropy ceramic blocks obtained by sintering have smaller grain sizes in W-HECs-1900, with an average grain size of 2.7 μm in D-HECs-1900 and 1.8 μm in W-HECs-1900.
3. In multiphase ceramics with SiC particles as the second phase toughening, the toughening mechanism is the result of the joint action of various mechanisms, such as microcracking, deflection and crack bridging. The Vickers hardness of D-HECs-1900 is HV_0.1_ = 1855.874 kg/mm^2,^ and the fracture toughness is K_IC_ = 4.154 MPa · m^1/2^. The Vickers hardness of W-HECs-1900 is HV_0.1_ = 2178.409 kg/mm^2^, and the fracture toughness is K_IC_ = 4.421 MPa · m^1/2^.
4. W-HECs-1900 has higher friction coefficient and smaller wear rate than D-HECs-1900. The friction coefficient of D-HECs-1900 is 0.18, and the wear rate is 1.443 × 10^−7^ mm^3^·N^−1^·m^−1^. The friction coefficient of W-HECs-1900 is 0.22, and the wear rate is 1.007 × 10^−8^ mm^3^·N^−1^·m^−1^.

## Figures and Tables

**Table 1:** The composition of D-HECs-1900 to the 5 points in Figure 7a.

Element Content (at%)	C	O	Si	Ti	Cr	Zr	Nb
1	25.18	1.95	70.03	0.21	0.14	0.66	1.83
2	0.00	7.36	1.59	1.32	0.82	0.00	88.92
3	7.09	15.14	17.47	9.02	26.68	11.77	12.82
4	6.48	9.53	3.69	22.11	2.19	29.29	26.71
5	4.72	15.95	3.61	16.35	1.70	35.96	21.17

**Table 2:** The composition of W-HECs-1900 to the 6 points in Figure 7b.

Element Content (at%)	C	O	Si	Ti	Cr	Zr	Nb
6	7.63	10.15	10.80	16.91	3.79	22.48	28.24
7	13.91	4.73	58.57	5.37	0.73	8.17	8.51
8	6.40	13.32	25.35	3.06	9.42	9.19	33.26
9	7.97	8.55	16.09	16.26	1.94	20.60	28.59
10	5.97	10.31	10.33	14.07	2.61	30.41	26.30
11	6.23	29.07	2.88	8.31	1.63	39.02	12.86

**Table 3:** Grain structure information for carbide raw materials.

	Carbides	Cr_3_C_2_	ZrC	NbC	TiC	α-SiC	β-SiC
	Space Group	Pnma	$\mathrm{Fm}\bar{3}$m	$\mathrm{Fm}\bar{3}$m	$\mathrm{Fm}\bar{3}$m	P6_3_mc	$F\bar{4}$3m
Our work	a (Å)	5.524	4.699	4.469	4.327	3.081	4.348
	b (Å)	2.828	4.699	4.469	4.327	3.081	4.348
	c (Å)	11.466	4.699	4.469	4.327	15.117	4.348
Other work	a (Å)	5.480	4.711	4.483	4.331		4.057
	b (Å)	2.786	4.711	4.483	4.331		4.057
	c (Å)	11.461	4.711	4.483	4.331		4.057

**Table 4:** The average atomic size difference of high entropy carbide solid solution that can be formed.

		Components	δ_a_ (%)	δ_b_ (%)	δ_c_ (%)
Our work	(TiCrZrNbSi) C_5_	α-SiC	13.62	18.86	54.07
	(TiCrZrNbSi) C_5_	β-SiC	9.7	17.31	48.32
	(TiCrZrNb) C_4_		9.7	18.02	48.39
Other work	(TiCrZrNbSi) C	β-SiC	9.9	17.8	48.6

**Table 5:** Sintering process, density and grain size of high entropy carbide ceramics by spark plasma sintering.

Ceramic Compositions	Sintering Process	Density	Average Grain Size (µm)	Reference
(Hf_0.2_Zr_0.2_Ta_0.2_Nb_0.2_Ti_0.2_)C	SPS/2000 °C/30 MPa/5 min	95%	16.5 ± 4.2	[24]
(Zr_0.25_Ti_0.25_Nb_0.25_Ta_0.25_)C	SPS/2000 °C/50 MPa/5 min	99%	10.3	[25]
(Ti_0.2_Nb_0.2_Ta_0.2_Hf_0.2_V_0.2_)C	SPS/2000 °C/50 MPa/10 min	99%	11	[25]
(Ti_0.25_V_0.25_Nb_0.25_Ta_0.25_)C	SPS/2100 °C/30 MPa/10 min	96%	6.72	[26]
(Hf_0.25_Ta_0.25_Zr_0.25_Nb_0.25_)C	SPS/2300 °C/40 MPa/7 min	98%	6.6 ± 3.6	[28]
(Hf_0.2_Mo_0.2_Nb_0.2_Ta_0.2_Ti_0.2_)C	SPS/1900 °C/40 MPa/10 min	96%	1.7 ± 0.6	[29]
(Nb_0.25_Ta_0.25_Mo_0.25_W_0.25_)C	SPS/1800 °C/30 MPa/20 min	97%	9 ± 0.5	[30]
(V_0.2_Nb_0.2_Ta_0.2_Mo_0.2_W_0.2_)C	SPS/1850 °C/30 MPa/20 min	98%	10	[31]
D-HECs-1900	SPS/1900 °C/50 MPa/15 min	92%	2.7	Our work
W-HECs-1900	SPS/1900 °C/50 MPa/15 min	96%	1.8	Our work

**Table 6:** The composition of fracture to the 2 points in Figure 11.

Element Content (at%)	C	O	Si	Ti	Cr	Zr	Nb
1	85.86	2.79	0.66	0.47	1.29	5.37	3.78
2	90.35	2.54	1.89	1.35	0.13	2.15	1.58

**Table 7:** Fracture toughness of high-entropy carbide ceramics.

Ceramic Compositions	Load (N)	Fracture Toughness (MPa·m^1/2^)	Testing Method	Reference
(Ti_0.25_V_0.25_Nb_0.25_Ta_0.25_)C	9.807	3.08	Vickers indentation	[26]
(Ta_1/3_Zr_1/3_Nb_1/3_)C	9.807	3.07	Vickers indentation	[33]
(Ta_0.25_Zr_0.25_Nb_0.25_Hf_0.25_)C	9.807	3.79	Vickers indentation	[33]
(Ta_0.25_Zr_0.25_Nb_0.25_W_0.25_)C	9.807	4.02	Vickers indentation	[33]
(Ta_0.2_Zr_0.2_Nb_0.2_Hf_0.2_W_0.2_)C	9.807	3.48	Vickers indentation	[33]
(Hf_0.2_Zr_0.2_Ta_0.2_Nb_0.2_Ti_0.2_)C	19.613	3.0 ± 0.2	Vickers indentation	[34]
(Ti_0.2_Zr_0.2_Nb_0.2_Ta_0.2_Mo_0.2_)C	98.067	3.28 ± 0.12	Vickers indentation	[35]
(Ta_0.2_Nb_0.2_Ti_0.2_V_0.2_W_0.2_)C	4.903	3.4	Vickers indentation	[36]
D-HECs-1900	4.903	4.15	Vickers indentation	Our work
W-HECs-1900	4.903	4.42	Vickers indentation	Our work

**Table 8:** The composition of friction layer to the 3 points in Figure 13.

Element Content (at%)	C	O	Si	Ti	Cr	Zr	Nb
1	8.84	39.85	12.05	9.15	0.52	15.99	13.24
2	8.03	39.40	9.32	10.77	1.10	16.80	14.59
3	8.66	28.09	5.36	14.90	1.20	20.54	21.25

## Data Availability

The original contributions presented in this study are included in the article. Further inquiries can be directed to the corresponding author.
